# Traumatic Brain Injuries Impact on School One Month and One Year After Injury

**DOI:** 10.1089/neur.2022.0069

**Published:** 2023-08-10

**Authors:** Erik B. Philipson, Joan Machamer, Sureyya Dikmen, Nancy Temkin

**Affiliations:** ^1^ Harborview Injury Prevention and Research Center, University of Washington, Seattle, Washington, USA.; ^2^ Department of Neurological Surgery, University of Washington, Seattle, Washington, USA.; ^3^ Department of Rehabilitation Medicine, University of Washington, Seattle, Washington, USA.; ^4^ Department of Biostatistics, University of Washington, Seattle, Washington, USA.

**Keywords:** concussion, return to learn, school health, traumatic brain injury

## Abstract

Traumatic brain injury (TBI) is a major cause of death and disability among the American population, but the impact TBIs have on the school experience of high school, and post-secondary students, is poorly understood. In this study, a cohort of 79 students, ages 15–22, with mild-to-severe TBIs, were retrospectively identified from the University of Washington Traumatic Brain Injury Database and Sample Repository. The Sickness Impact Profile (SIP) was used to determine the frequency at which schooling was impacted by a TBI and identify the most common self-reported issues students faced in their return to school. At 1 month post-injury, 70% of students either had not returned to school as a result of their TBI or had returned to school but experienced issues related to their TBI. The most-reported issues at 1 month were a difficulty keeping up with school work as a result of it taking longer to complete assignments, tiring easily, having to take frequent rests, and grades that were not as good as they used to be. At 1 year post-injury, the number of students whose TBIs were affecting their school situation dropped 20 percentage points to 49%. The most reported issues at 1 year were forgetting more quickly what was learned in class and having more difficulty understanding new concepts and material. These findings indicate that TBIs have a profound effect on a student's school experiences up to at least 1 year post-injury.

## Introduction

Each year in the United States, there are ∼50,000 traumatic brain injury (TBI)-associated hospitalizations among youth age ≤17 years.^1^ These hospitalizations range in severity from the most severe TBI to mild TBIs, or concussions. Those with more severe TBIs as well as youth with concussions are known to experience physical, cognitive, sleep, and emotional symptoms that may affect their ability to function as students within a traditional classroom environment.^2–5^

Although those diagnosed with a TBI have access to formal support in their return to the classroom under the Individuals with Disabilities Education Act (IDEA) law and may utilize an Individualized Educational Plan (IEP), the current system of care has gaps that often lead to students not receiving the resources they need to make a successful return.^6^ At the same time, many students with concussion experience difficulty upon return to school, and those who return to the classroom without support and remain out of school for an extended period of time are known to report longer lasting symptoms than those who are supported by a formal return to learn (RTL) process.^7–10^ In response to this, using input from health professionals, policy makers have implemented legislation and programs, such as the well-known return to play protocol and public health campaign, to lessen the burden faced by young adults who sustain a concussion as they return to participating in sports.^11,12^ In light of its success, the return to play system has been used as a springboard to develop and implement RTL programs, which are aimed at facilitating a smooth, healthy return to the classroom after a concussion.^13–17^

Recent work has shown that implementing a student-centered RTL care plan is feasible and acceptable in public high schools for those returning to the classroom after a concussion.^18^ Use of an RTL care plan is also reported to facilitate the RTL of students with a concussion and may be able to aid in the identification of students who need additional support.^19^ That said, the use of an RTL care plan remains untested in populations of those with more severe TBIs, and current RTL guidelines are primarily based on our understanding of concussions as they present in a clinical environment; there is little research that examines the impact of a concussion, or more severe TBI, directly on a student's school experience as reported by the students themselves.^20^ Research into the types of school-specific issues faced by those returning to the classroom after a TBI is needed to develop more effective, further optimized RTL programs.

This study expands on the current literature by investigating students' self-reported experiences as they return to school after TBIs of all severity levels. Specifically, this study aims to determine how frequently TBIs of all severity levels impact a student's school experience at 1 month and 1 year post-injury and the types of issues students returning to school after a TBI report as impacting their schooling. The results of this study will better the understanding of students' classroom-specific experiences after a TBI, which will promote the further development and optimization of RTL care plans.

## Methods

This study was submitted to and approved by the University of Washington Institutional Review Board (UW IRB). The UW IRB also approves the data repository from which subjects were identified.

### Participants

Participants for this study were retrospectively identified from the University of Washington Traumatic Brain Injury Database and Sample Repository. Participants included in this study were originally recruited to participate in one of the following studies: Valproate Prophylaxis of Post-traumatic Seizures with participant enrollment from 1991 to 1995; Dilantin Prophylaxis of Post-traumatic Seizures with participant enrollment from 1983 to 1987; Patient Characteristics and Head Injury Outcomes with participant enrollment from 1984 to 1986; and Behavioral Outcome of Head Injury with participant enrollment from 1980 to 1982.^21–24^ To be included in this study, participants also had to state that they were a student and were between 15 and 22 years of age at the time of their injury. All of the participants in this study, including those with mild TBI, were hospitalized. Participants also had to have completed the school subsection of the Sickness Impact Profile (SIP), as modified by Temkin and colleagues, at 1-month and 1-year follow-up visits.^25^ In all, 114 students were identified from the four repository data sets. Of these 114 students, 28 were missing data from 1 month post-injury and 7 were missing data from 1 year post-injury. This left 79 participants who met the inclusion criteria and were included in the analysis for the present article (Supplementary Fig. S1).

### Measures

The school subtest of the modified SIP was used to examine the effects of TBI on schooling.^25^ The SIP is able to distinguish patients with TBI from comparison subjects and is a validated metric for those with TBI.^25^ All of the subjects in this study completed the school subsection of the SIP, at both their 1-month and 1-year injury follow-up visits. The school section of the SIP is a structured outcome questionnaire administered by study personnel that consists of three yes or no questions and 21 statements that participants can endorse if they feel it describes them (Supplementary Appendix S1).^25^

Participants were instructed to respond to the statements based upon their status on the day of the interview, unless the interview took place during the weekend—in which case they were to respond as if it were a school day. Participants score a 0 if they indicate that they either returned to school with no issues or had not returned to school for reasons unrelated to their TBI. Participants score a 1 if they indicate that they have not returned to school because of their TBI. Each of the 21 statements has a weight and the score is the sum of the weights, yielding a value between 0 and 1. In this study, subjects' answers to the questions and statements on the school portion of the SIP were used to determine the frequency with which TBIs impacted subjects' daily school functioning and identify the most common types of issues that students encountered.

Scores on the school portion of the SIP range from 0 to 1, with a score of 0 indicating that a participant's TBI or health had no impact on their school functions and a score of 1 indicating it had a maximal impact^15^; all scores >0 indicate that a subject's TBI impacted their school functioning. Participants were divided into two analysis groups based upon their scores. Participants who scored a 0 were placed into the “No TBI Impact on School” group, and subjects who scored >0 were placed into the “TBI impact on School” group. Participants responded to statements based upon their status on the day of SIP administration, or as if it were a school day if it was the weekend. The most common issues upon return to school were identified through examination of the items endorsed on the school portion of the SIP by subjects who scored between a 0 and a 1.

### Brain injury severity

Subjects initial post-resuscitation Glasgow Coma Scale (GCS) score was used as the measure of TBI severity. The GCS describes the level of consciousness in TBI patients through the summation of scores in three categories: eye-opening, motor response, and verbal response.^26^ GCS scores range from 3 to 15, with higher scores indicating a less severe TBI; scores of 13–15 indicate a mild TBI, 9–12 a moderate TBI, and 3–8 a severe TBI.^27^ For some analyses, those with GCS 13–15 were subdivided based on whether they had intracranial abnormalities on computed tomography (CT) imaging. Skull fracture or intracranial air were not counted as intracranial abnormalities.

### Demographics

The primary demographic variables in this study were age at the time of injury, sex, race, type of school program, injury circumstance, and pre-existing condition. A participant was classified as having a pre-existing condition if they had one or more of the following: a pre-existing alcohol condition, psychiatric condition, or central nervous system disorder. Pre-existing alcohol conditions were defined as pre-injury treatment for alcohol problems, including Alcoholics Anonymous (AA), inpatient alcohol treatment program, outpatient program, or similar. Pre-existing psychiatric conditions were defined as a serious psychiatric condition, for example, inpatient psychiatric hospitalization and/or major diagnosis (schizophrenia, manic-depressive illness, or suicide attempt) or long-term use of antidepressant medication.

### Statistical analysis

Fisher's exact or chi-square tests were used to compare categorical variables among groups. Analyses of variance were used to compare continuous variables. All of the analyses was conducted using IBM SPSS Statistics software (version 25; IBM Corp., Armonk, NY).

## Results

Demographic and injury characteristics of the 79 subjects included in this study are shown in Table 1. Participants were primarily male (63%), 15–17 years of age (51%), mostly white (90%), and attending a high school at the time of their injury (58%). Pre-existing conditions were present in 16.5% of participants. The majority of participants had mild TBIs (76%). All of the 79 subjects were included in both the 1-month and 1-year impact analyses.

**Table 1.** Demographics and Injury Characteristics of Study Participants

Demographics and injury characteristics of study participants	Students* *(*N* = 79)*N *(%)
Age (years)	
15–17	40 (50.6)
18–20	30 (38)
21–22	9 (11.3)
Sex	
Male	50 (63.3)
Female	29 (36.7)
Race	
White	71 (89.9)
African American	6 (7.6)
Other	2 (2.5)
Pre-existing condition(s)	
Present	13 (16.5)
School program	
High school	45 (57.7)
4-year college	9 (11.5)
Community college/tech	13 (16.7)
Other	11 (14)
Injury circumstance	
Motor vehicle accident	45 (57)
Bike accident	6 (7.6)
Pedestrian	8 (10.1)
Fall	9 (11.4)
Assault	5 (6.3)
Other	6 (7.6)
TBI severity	
Mild (GCS 13–15)	60 (75.9)
CT abnormality	33 (55.0)
No CT abnormality	20 (33.3)
Unknown CT status	7 (11.7)
Moderate (GCS 9–12)	14 (17.7)
Severe (GCS 3–8)	5 (6.3)

All of the information in this table is reported as *N* (%) and includes the entire study population.

Tech, technical college; GCS, Glasgow Coma Scale; CT, computed tomography scan.

The frequency at which a TBI impacted subjects' schooling within the entire study group is examined in Table 2. At 1 month post-injury, 55 participants, 70% of the study group's schooling, were impacted by their TBI, with 46% of the group having not returned to school at all as a result of their injury and 24% returning to school but with issues. One year after injury, the percentage of the entire study group still reporting that the TBI impacted their schooling fell to 49.4%. The number of participants who had not returned because of their injury dropped to 8.9%, but the number reporting a return with issues grew to 40.5%. No significant differences were observed between participates with pre-existing conditions compared to those without.

**Table 2.** School Outcomes Stratified by Injury Severity and CT Status

Panel A: 1-month school outcomes stratified by CT status	1 month					
	Entire study population (*N* = 79)*N *(%)	All moderate and severe TBI	Mild TBI			
			All mild TBI	CT abnormal	CT normal	CT Unk.
		(*N* = 19)	(*N* = 60)	(*N* = 33)	(*N* = 20)	(*N* = 7)
		*N *(%)	*N *(%)	*N *(%)	*N *(%)	*N *(%)
**No Impact on School**	**24 (30.4)**	**4 (21.1)**	**20 (33.3)**	**10 (30.3)**	**7 (35.0)**	**3 (42.9)**
Has Returned–No Issues Reported	11 (14.0)	2 (10.5)	9 (15.0)	3 (9.1)	5 (25.0)	1 (14.3)
Has Not Returned–Unrelated to Injury	13 (16.5)	2 (10.5)	11 (18.3)	7 (21.2)	2 (10.0)	2 (28.6)
**School Impacted**	**55 (69.6)**	**15 (78.9)**	**40 (66.7)**	**23 (69.7)**	**13 (65.0)**	**4 (57.1)**
Has Returned–Reports Issues	19 (24.1)	3 (15.7)	16 (26.7)	11 (33.3)	5 (25.0)	0 (0.0)
Has Not Returned–Injury Related	36 (45.6)	12 (63.2)	24 (40.0)	12 (36.4)	8 (40.0)	4 (57.1)

Panel B: 1-year school outcomes stratified by CT status	1 year					
**No Impact on School**	**40 (50.6)**	**4 (21.1)**	**36 (60.0)**	**22 (66.7)**	**10 (50.0)**	**4 (57.1)**
Has Returned–No Issues Reported	35 (44.3)	4 (21.1)	31 (51.7)	18 (54.5)	9 (45.0)	4 (57.1)
Has Not Returned–Unrelated to Injury	5 (6.3)	0 (0.0)	5 (8.3)	4 (12.1)	1 (5.0)	0 (0.0)
**School Impacted**	**39 (49.4)**	**15 (78.9)**	**24 (40.0)**	**11 (33.3)**	**10 (50.0)**	**3 (42.9)**
Has Returned–Reports Issues	32 (40.5)	13 (68.4)	19 (31.7)	7 (21.2)	9 (45.0)	3 (42.9)
Has Not Returned–Injury Related	7 (8.9)	2 (10.5)	5 (8.3)	4 (12.1)	1 (5.0)	0 (0.0)

This table is split into two panels, entitled Panel A and Panel B. Panel A contains school outcomes at 1 month, and Panel B contains school outcomes at 1 year. All categories are reported as *N* (%) for columns within their respective panels. Subjects in the *No Impact on School* group are those whose Sickness Impact Profile (SIP) score was 0. This group is subdivided by whether they had returned to school. Subjects in the *School Impacted* group are those whose SIP score was >0. Subjects whose scores were <1 but >0 were placed into the *Has Returned–Reports Issues* group, and subjects whose score was 1 were placed into the *Has Not Returned–Injury Related* group.

CT, computed tomography scan; Unk., unknown; TBI, traumatic brain injury; Mod., moderate; Sev., severe.

Table 2 also examines the frequency at which participants' schooling was impacted by TBI severity—moderate and severe injuries are combined into one group, and the mild TBI group is presented as a whole and also subdivided by the presence/absence of intracranial CT abnormal findings. Overall, at 1 month post-injury, 67% of participants in the mild TBI group and 79% of participants in the combined moderate and severe group reported an impact on their schooling. At 1 year, 40% of participants in the mild TBI group and 79% of participants in the combined moderate and severe group reported an impact on their schooling. Within the GCS mild TBI group, 55% of participants had intracranial CT abnormalities (complicated mild cases). One month post-injury, 70% of participants with abnormal, and 65.0% of participants with normal, intracranial CT findings reported an impact on their schooling. One year post-injury, 33.3% of participants with abnormal, and 50% of participants with normal, intracranial CT findings continued to report an impact on their schooling. At both 1 month and 1 year post-injury, a higher proportion of the moderate/severe TBI group reported not returning to school because of their injuries than the mild TBI group (1 month, 63.2% vs. 40.0%; 1 year, 10.5% vs. 8.3%).

Comparisons between participants in the “School Impacted” or “School Not Impacted” groups at the 1-month and 1-year follow-up time points on demographic characteristics and TBI severity are found in Table 3. At 1 month, there was no difference between groups with respect to TBI severity, school program, pre-existing conditions, sex, or age. However, at the 1-year follow-up, there was a significant difference between the two groups in terms of sex (χ^2^ = 4.781, *p* = 0.029) and TBI severity (*p* = 0.011, Fisher's exact test)—more females reported an impact, and TBI severity was greater in the group that experienced difficulties at school.

**Table 3.** Comparison of Subjects Whose School Function Was Impacted vs. Not Impacted

Comparison of subjects whose school function was impacted vs. not impacted	1 month			1 year		
	Impacted	Not impacted	*p *value	Impacted	Not impacted	*p *value
*n*	55	24		39	40	
Age, mean (SD)	18 (2.0)	18 (2.0)	0.221	18 (2.0)	18 (2.0)	0.389
Sex, *n* (%)			0.546			0.029^*^
Male	36 (72.0)	14 (28.0)		20 (40.0)	30 (60.0)	
Female	19 (65.5)	10 (34.5)		19 (65.5)	10 (34.5)	
Pre-existing condition, *n* (%)			0.520			0.337
Present	8 (61.5)	5 (38.5)		8 (61.5)	5 (38.5)	
Absent	47 (71.2)	19 (28.8)		31 (47.0)	35 (53.0)	
School program, *n* (%)			0.634			0.665
High school	33 (73.3)	12 (26.7)		22 (48.9)	23 (51.1)	
4-year college	5 (55.6)	4 (44.4)		5 (55.6)	4 (44.4)	
Com./Tech. college	10 (76.9)	3 (23.1)		5 (38.5)	8 (61.5)	
Other	7 (63.6)	4 (36.4)		7 (63.6)	4 (36.4)	
TBI severity, *n* (%)			0.679			0.011^*^
Mild (GCS 13–15)	40 (66.7)	20 (33.3)		24 (40.0)	36 (60.0)	
CT abnormality	23 (69.7)	10 (30.3)		11 (33.3)	22 (66.7)	
No CT abnormality	13 (65.0)	7 (35.0)		10 (50.0)	10 (50.0)	
Unknown CT status	4 (57.1)	3 (42.9)		3 (42.9)	4 (57.1)	
Moderate (GCS 9–12)	11 (78.6)	3 (21.4)		11 (78.6)	3 (21.4)	
Severe (GCS 3–8)	4 (80.0)	1 (20.0)		4 (80.0)	1 (20.0)	
						

Age and GCS are reported for each group as mean (SD). Sex, pre-existing condition, school program, and TBI severity are reported for each group as *N* (%), where the percentage is the row percent for the group at the given time point. Fisher's exact tests were used to calculate the *p* values for categorical variables.

^*^ Significant at an alpha level of 0.05.

SD, standard deviation; Com., community college; Tech., technical college; TBI, traumatic brain injury; GCS, Glasgow Coma Scale; CT, computed tomography.

The most common issues among the subgroup of participants returning to school and reporting difficulties at 1 month post-injury are reported in Table 4. Two issues—“I have difficulty keeping up with my school work…” and “My grades are not as good as they used to be”—were tied for the most frequently endorsed, at 42%. The most common issues experienced by those returning to school with difficulties at the 1-year follow-up are reported in Table 5. The majority of students (59%) at this follow-up reported that they forget more quickly what they learn in class. Additionally, 50% reported having more difficulty understanding new concepts and material.

**Table 4.** Most Reported School Issues at 1 Month Post-Injury

Most reported school issues at 1 month post-injury	1 month (*N* = 19)*N *(%)
I have difficulty keeping up with my school work, for example, it takes me longer to complete my assignments, I tire easily and have to take frequent rests.	8 (42.1)
My grades are not as good as they used to be.	8 (42.1)
I do my school work more slowly.	7 (36.8)
I have difficulty keeping up when a lot of reading is required for a class.	6 (31.6)
I am going to school for shorter hours or taking fewer classes.	5 (26.3)
I do not do my school work as well as I did before.	5 (26.3)

This table depicts the most reported school issues among participants who returned to school but are experiencing issues at 1 month post-injury. All categories are reported as *N* (percentage of those reporting issues).

**Table 5.** Most Reported School Issues at 1 Year Post-Injury

Most reported school issues at 1 year post-injury	1 year (*N* = 32)*N *(%)
I forget more quickly what I learn in class.	19 (59.4)
I have more difficulty understanding new concepts and material.	16 (50.0)
I have difficulty keeping up with my school work, for example, it takes me longer to complete my assignments, I tire easily and have to take frequent rests.	14 (43.8)
My grades are not as good as they used to be.	14 (43.8)
I do my school work more slowly.	13 (40.6)
I do not do my school work as well as I did before.	13 (40.6)

This table depicts the most reported school issues among participants who returned to school but are experiencing issues at 1 year post-injury. All categories are reported as *N* (%).

## Discussion

This study builds on the current literature that has explored the numerous effects of TBIs on young adults by determining the frequency at which an injury-severity–diverse cohort is impacted in their school lives by a TBI and the specific types of issues these students face at 1 month and 1 year post-injury. The results of this study provide the information needed to help drive the creation of more effective RTL programs across the country that can help all TBI survivors. It will also help to combat the incorrect belief of many teachers and professors that after 1 month a student should either have completely returned to full functioning or have had an injury that is too severe for them to return to a regular classroom.

This study found that it is more likely for a student's school life to be impacted by a TBI at 1 month post-injury than not. This finding held true both within the entire population (70% impacted) and within the mild subset (67% impacted) and was the expected outcome based upon the results of studies that examined various outcomes in similar demographic groups.^4,28,29^ These numbers may be slightly lower than reality given that there were 13 students at the 1-month follow-up who reported that they had not returned to school for reasons unrelated to their injury. However, 8 of the 13 were on summer break at the time of the interview and may have underestimated the impact of their TBI on school functioning because of lack of experience with return to school. Further, the results of this study indicate that at 1 month after injury, a large subset of participants (46%) have not yet even returned to school as a result of their injuries.

In fact, this study found that only 38% of students returned to school within 1 month after injury, the majority of those with issues. This is a novel finding that indicates that an RTL time frame that instills the belief that all students with a TBI will be back to class and function at normal levels within 1 month after the injury is much too fast and may hurt many students. This point is further supported by the fact that at 1 month post-injury, this study found no statistically significant difference in school impact among TBI severity levels.

At 1 year post-injury, this study found that 85% of students had returned to school, a higher rate than found in another study that examined the 1- to 10-year frequency of return to school.^30^ If taken at face value, this study's 1-year return to school frequency appears to indicate relatively good school outcomes. However, when examined further, it is revealed that 41% of the population who returned to school encountered issues and 9% of the population had yet to return to school at all. These numbers dropped slightly among the mild group to 32% and 8%, respectively, and there was a statistically significant difference in school impact between TBI severity levels. Even though the rates observed in this study are slightly lower within the mild TBI population, given the estimated number of mild TBI cases in the United States each year, there is the potential that a large number of persons could be affected.

That said, this study has limitations that could affect the generalizability of the results—all of the participants in this study were admitted to the hospital, and, within the mild TBI group, CT abnormalities were present in a larger percentage of participants than the epidemiology suggests. Considering this, it is unclear how the findings of this article will generalize to the larger TBI population where, especially for those with GCS 13–15, many are discharged home from the emergency department and CT abnormalities are present in fewer cases.^31,32^ When considering how this affects how well the results of this study may generalize, it is important to note, in this study, that those without CT abnormalities reported difficulties at similar rates to those with mild TBI with CT abnormalities. This study was also limited in the conclusion that can be made because it lacked any trauma-control group, relied upon self-reporting, used data from studies conducted many years ago, and, as a retrospective analysis, was bound by the number and types of questions that were asked of the subjects upon their enrollment into other studies.

It is also important to consider that because this study was limited to those who completed the SIP at both 1 month and 1 year, it may underestimate difficulties in those with more severe injuries, given that cases in which participants were unable to take the SIP at 1 month because of impairment were excluded, potentially resulting in a bias toward a higher return to school frequency. Nevertheless, the results of this study indicate that the true effect of TBI on school at 1 year has been hiding behind a high return to school frequency that looks good on the surface, but, in fact, includes numerous students who are struggling as a result of their TBI. This finding indicates a need for students who are returning to school after a TBI to be followed closely by teachers, administrators, and family members for at least a year after their injury to ensure that they receive proper accommodation and that their return is successful.

Students who had returned to school 1 month post-injury and faced issues reported difficulties that mostly had to do with their inability to complete the same amount of work in the same time frame as they did pre-injury and their ability to process and retain new information.

For many, problems were sufficient to have a perceived impact on grades. Given that there is not much schools and loved ones can do about helping a student's brain return to baseline after TBI, it is vital that they work with students to implement accommodations that can help to mitigate these issues beyond just the reduced course schedule that 26% of the students returning to school with issues reported having.

At 1 year post-injury, among students who had returned to school but faced difficulties, the two most reported issues had to do with their functioning levels; students report forgetting more quickly what they learned in class and having more difficulty understanding new concepts and material. Students also continued to frequently report that they struggled to keep up with their school work and were doing their work more slowly and that their performance in school was not as good as it has been in the past. The identification of these issues indicates that schools need to continue to work with students who have returned from a TBI to implement appropriate accommodations for more than a year after their injury.

Although this study was able to identify the frequency with which students returning to school after a TBI face issues and the most common types of issues reported by those students, it is unable to make suggestions about what types of accommodations or policies could help to mitigate the issues and increase the number of successful issue-free returns to the classroom. Thus, there is a need for future studies to continue to examine the array of issues experienced by those with TBI upon their return to school and the types of accommodations or policies that may best be able to address them.

## Supplementary Material

Supplemental data

Supplemental data

## Abbreviations Used

CT: computed tomography
GCS: Glasgow Coma Scale
RTL: return to learn
SIP: Sickness Impact Profile
TBI: traumatic brain injury
UW IRB: University of Washington Institutional Review Board
