# Supplementary figures and images for: Traumatic Brain Injuries Impact on School One Month and One Year After Injury
Source: Neurotrauma Rep. 2023 Aug 10;4(1):507–14. doi: 10.1089/neur.2022.0069 (PMC10523406; doi:10.1089/neur.2022.0069)

<FL>Supplementary Fig. S1: Flowchart for study inclusion criteria.


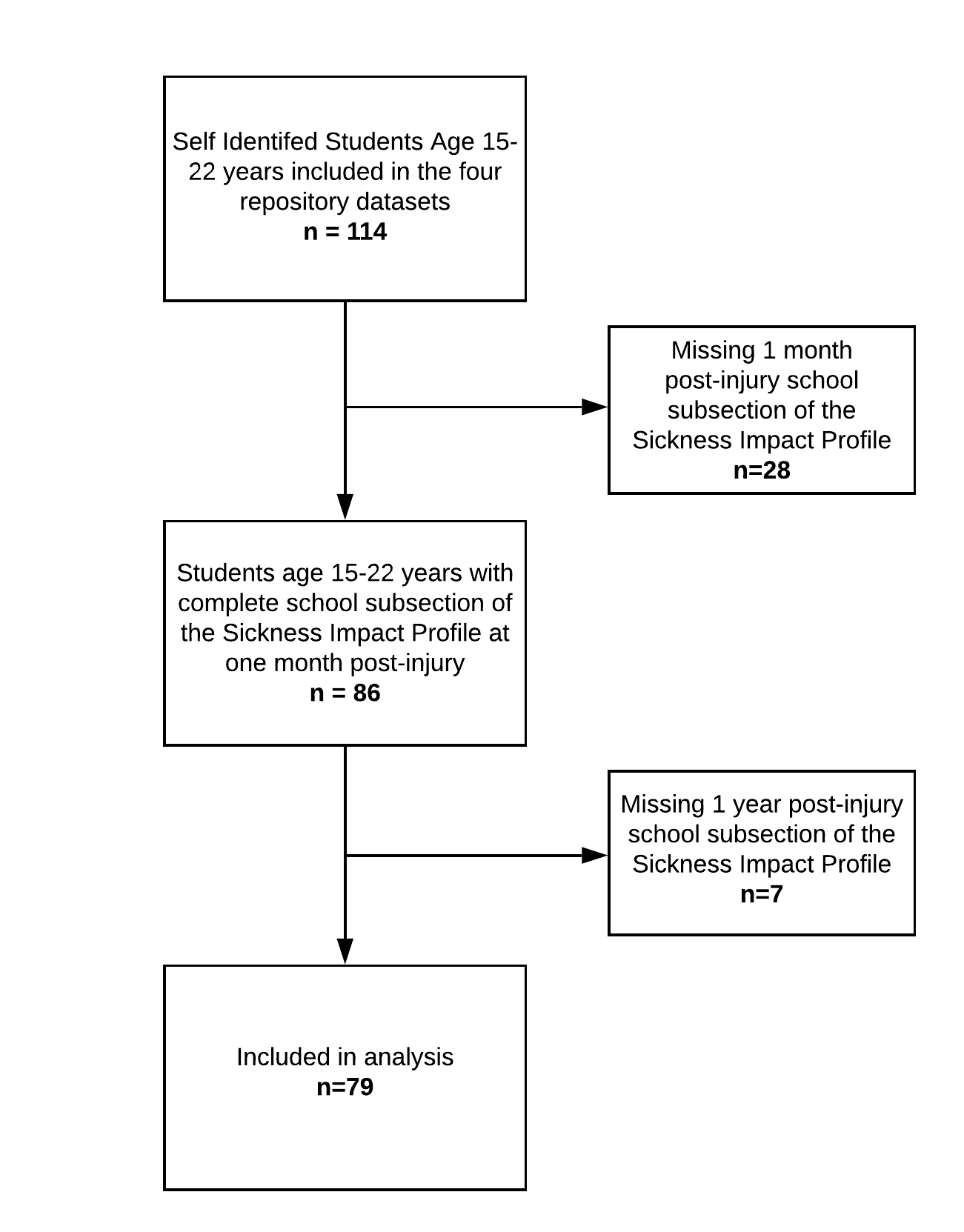

Supplement: Supplemental data [file Suppl_FigS1.docx]
