# Supplementary material for: Traumatic Brain Injuries Impact on School One Month and One Year After Injury
Source: Neurotrauma Rep. 2023 Aug 10;4(1):507–14. doi: 10.1089/neur.2022.0069 (PMC10523406; doi:10.1089/neur.2022.0069)
Supplement: Supplemental data [file Suppl_AppendixS1.docx]

**Supplemental Digital Content – Appendix 1:** Sickness Impact Profile School Section Questions

Yes / No Questions

1. Were you enrolled in school a month/year ago?
2. have you attended school in the past month/year?
   1. if you answered no, you have not attended school in the past month/year, is this related to your health or an injury?

Questions are endorsed that describe the participant today and are related to his/her state of health or injury:

1. I am not going to school or receiving any schooling.
2. I have different educational plans.
3. I am not going to school but am being tutored.
4. I am going to school for shorter hours or taking fewer classes.
5. I am going to school but am now taking special classes.
6. I am going to school because I have different vocational plans.
7. I have difficulty keeping up with my school work, for example, it takes me longer to complete my assignments, I tire easily and have to take frequent rests.
8. I do my school work more slowly.
9. My grades are not as good as they used to be.
10. I do not do my school work as carefully and accurately.
11. I don’t get along as well as I used to with my schoolmates and teachers.
12. I have more difficulty understanding new concepts and material.
13. I forget more quickly what I learn in class.
14. I can’t do as well as I used to when a class requires a lot of written reports or term papers.
15. I have difficulty with math or arithmetic computations.
16. I have difficulty keeping up when a lot of reading is required for a class.
17. I frequently ask for help with my school work.
18. I do not do my school work as well as I did before.
19. I do better in my school work when someone tells me exactly what to do.
20. Sometimes I become confused even when I am doing things I used to do without difficulty.
21. I no longer do my school work in a confident way.
